# Cytogenetic and genomic organization analyses of chloroplast DNA invasions in the nuclear genome of *Asparagus officinalis* L. provides signatures of evolutionary complexity and informativity in sex chromosome evolution

**DOI:** 10.1186/s12870-019-1975-8

**Published:** 2019-08-16

**Authors:** Shu-Fen Li, Jia-Rong Li, Jin Wang, Ran Dong, Ke-Li Jia, Hong-Wei Zhu, Ning Li, Jin-Hong Yuan, Chuan-Liang Deng, Wu-Jun Gao

**Affiliations:** 1 College of Life Sciences, Henan Normal University, Xinxiang, 453007 China; 2 SanQuan Medical College, Xinxiang Medical University, Xinxiang, 453003 China

**Keywords:** Chloroplast DNA, *Asparagus officinalis*, Male-specific region of Y chromosome (MSY), Nuclear integrants of plastid DNA (NUPTs), Sex chromosome

## Abstract

**Background:**

The transfer of chloroplast DNA into nuclear genome is a common process in plants. These transfers form nuclear integrants of plastid DNAs (NUPTs), which are thought to be driving forces in genome evolution, including sex chromosome evolution. In this study, NUPTs in the genome of a dioecious plant *Asparagus officinalis* L. were systematically analyzed, in order to investigate the characteristics of NUPTs in the nuclear genome and the relationship between NUPTs and sex chromosome evolution in this species.

**Results:**

A total of 3155 NUPT insertions were detected, and they represented approximated 0.06% of the nuclear genome. About 45% of the NUPTs were organized in clusters. These clusters were derived from various evolutionary events. The Y chromosome contained the highest number and largest proportion of NUPTs, suggesting more accumulation of NUPTs on sex chromosomes. NUPTs were distributed widely in all of the chromosomes, and some regions preferred these insertions. The highest density of NUPTs was found in a 47 kb region in the Y chromosome; more than 75% of this region was occupied by NUPTs. Further cytogenetic and sequence alignment analysis revealed that this region was likely the centromeric region of the sex chromosomes. On the other hand, the male-specific region of the Y chromosome (MSY) and the adjacent regions did not have NUPT insertions.

**Conclusions:**

These results indicated that NUPTs were involved in shaping the genome of *A. officinalis* through complicated process. NUPTs may play important roles in the centromere shaping of the sex chromosomes of *A. officinalis*, but were not implicated in MSY formation.

**Electronic supplementary material:**

The online version of this article (10.1186/s12870-019-1975-8) contains supplementary material, which is available to authorized users.

## Background

In addition to nuclear genomes, two organellar genomes, namely, mitochondrial and plastid genomes, are found in plant cells. These three genomes coexist within each individual cell, where DNA is horizontally transferred between these cell parts, especially from the organelles to the nucleus [1, 2]. The extensive sequence transfer of organellar DNA into the nucleus produces nuclear integrants of plastid DNA (NUPTs) or nuclear integrants of mitochondrial DNAs (NUMTs). NUPTs and NUMTs provide new raw materials for genome complexity and remarkably contribute to chromosome evolution [3–6]. NUMTs exist widely in animals, plants, fungi, and protists [7, 8], whereas NUPTs only exist in plants. NUMTs have been extensively studied [9, 10], but NUPTs are still under research even though they have been explored in a number of plants [11].

NUPTs are widely distributed in plant species [8, 12]. Various properties, such as size, abundance, age, structure, and chromosomal localization of NUPTs have been analyzed in several model plant species [13, 14]. In *Arabidopsis*, approximately 18% of the total protein-coding genes are of chloroplast origin [13]. In *Oryza sativa*, large fragments of NUPTs are mainly distributed in the vicinity of centromeres. In addition, after plastid DNAs were integrated, these NUPTs were rapidly fragmented, shuffled, and most of them are discarded from the nuclear genome within a million years. These data in *O. sativa* indicate that the plant nuclear genome is in dynamic process of frequent integration and rapid elimination of the chloroplast genome and the pericentromeric regions are hot spots for the turnover of NUPTs [15]. The chromosomal distributions, correlation with transposable elements (TEs), and evolution dynamism of NUPTs show species-specific patterns. Although the integration mechanism of NUPTs is still not completely elucidated, non-homologous end joining and microhomology-mediated end joining of DNA double strand breaks are likely the mechanisms by which the organellar DNA fragments are integrated into the nucleus [16, 17]. NUPTs have also been cytogenetically analyzed in several plant species. For example, NUPT sites have been detected in 10 maize inbred lines through fluorescence in situ hybridization (FISH) analysis by using 14 overlapping fragments of the chloroplast genome. The integrations of NUPTs are recent and occurred frequently, as the distribution patterns vary greatly among different lines [18]. However, systematic analysis involving bioinformatics analysis and experimental cytogenetic studies in most plant species has yet to be performed.

Similar to TEs, inserted NUPTs can lead to the instability of the genome, such as causing chromosome structure rearrangement, size expansion, and heterochromatization [6, 19, 20]. Thus, the integration of NUPTs into the nuclear genome adds genetic diversity and is of physiological and evolutionary importance [21, 22]. Several studies revealed that NUPTs preferentially accumulate in the sex chromosomes of dioecious plants [23–25]. For example, in *Silene latifolia*, a bacterial artificial chromosome (BAC) clone containing a part of the chloroplast genome strongly hybridized to the Y chromosome, but its hybridization to the X chromosome and the autosomes is remarkably weak. The accumulation of these NUPTs may be one of the main factors of the size expansion of the Y chromosome in *S. latifolia* [23]. In another dioecious model plant, *Carica papaya*, NUPTs accumulated in the male-specific region of the Y chromosome (MSY) and the hermaphrodite-specific regions of Y^h^ (HSY) are 12 times more than those in the X chromosome and four times more than those of the genome-wide average. The difference is caused by the proliferation, usually co-amplifying with surrounding retrotransposons of NUPTs in the MSY and HSY [25]. These studies reveal that the accumulation of NUPTs is strongly related to the evolution of plant sex chromosomes.

Among dioecious plants, *Asparagus officinalis* is an economically and nutritionally important vegetable crop that is cultivated worldwide. It has a haploid genome size of 1308 Mb with 2*n* = 2*x* = 20 chromosomes [26, 27]. The sex type of *A. officinalis* is determined by a pair of homomorphic sex chromosomes, namely, X and Y. A small MSY with size of 847 kb is detected recently [28]. In addition, the YY genotype (‘supermale’) is also viable [29]. These features indicate that the sex chromosomes of *A. officinalis* are in an early stage of sex chromosome evolution. The young sex chromosomes of *A. officinalis* can be used as a useful model to characterize the early stages of sex chromosome evolution. The recently sequenced *A. officinalis* genome provides a unique opportunity for the systematic investigation of NUPTs throughout the genome. In this study, we analyzed the amount, distribution pattern, and age of NUPTs by applying bioinformatics methods and investigated the cytogenetic chromosome localization of NUPTs through FISH analysis. The obtained data could provide a valuable basis for further studying the genome structure and sex chromosome evolution of *A. officinalis*.

## Results

### Identification of NUPTs in the nuclear genome of *A. officinalis*

**Fig. 1:**
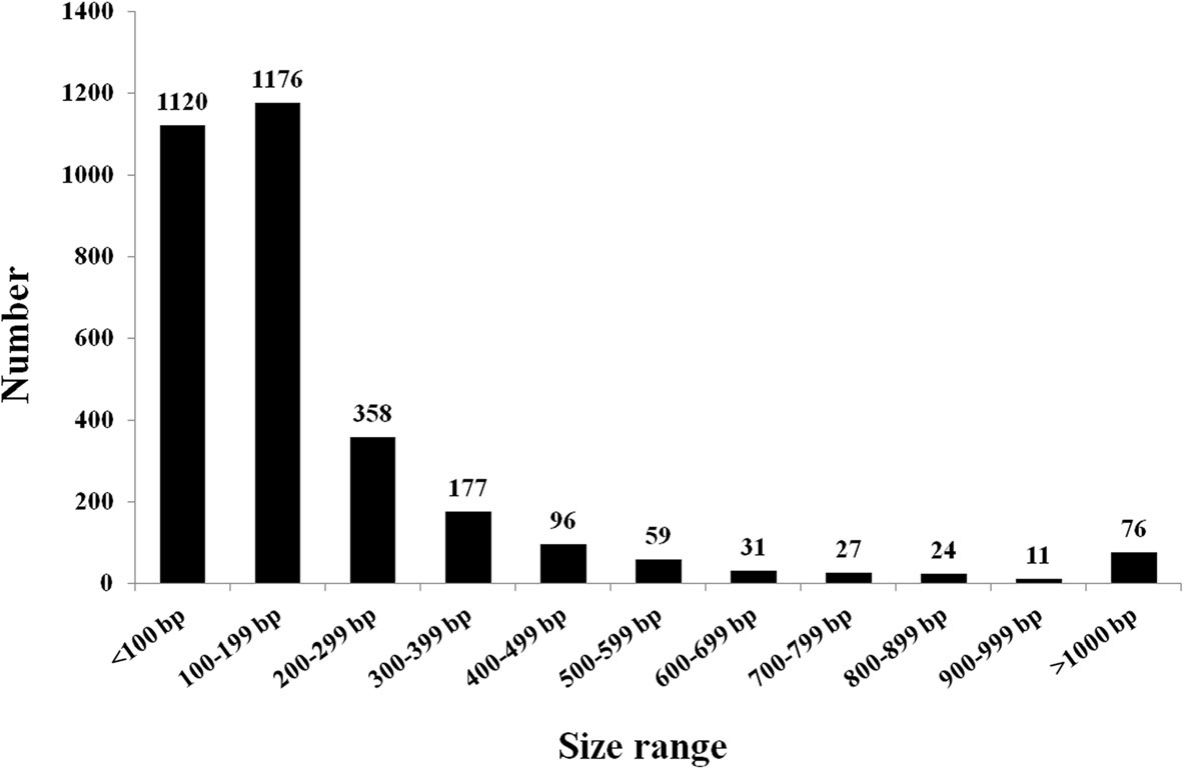
Size distribution of NUPTs in the nuclear genome of *A. officinalis*

We analyzed the chloroplast DNA sequences integrated in the nuclear genome of *A. officinalis*. A total of 3155 NUPT insertions were identified. The cumulative length of these NUPTs was 703,058 bp, constituting approximately 0.06% of the total nuclear genome. The identity of these matched nuclear sequences with the chloroplast DNA (cpDNA) sequence ranged from 71.81–100%. The NUPT sequence length varied from 33 to 8733 bp with a mean length of 223 bp (Additional file 1: Table S1). The distribution of NUPTs according to size is presented in Fig. 1. Generally, the number of NUPTs decreased sharply with increased size range. Approximately 72.8% of the NUPTs had a size of 33–199 bp, where 1120 small NUPTs are shorter than 100 bp and 1176 NUPTs had a size ranging from 100 to 199 bp. A total of 76 NUPTs (2.4%) had a large size of longer than 1000 bp. Among these long NUPTs, 19 are localized on chromosome 1, i.e. the Y chromosome [28].

**Fig. 2:**
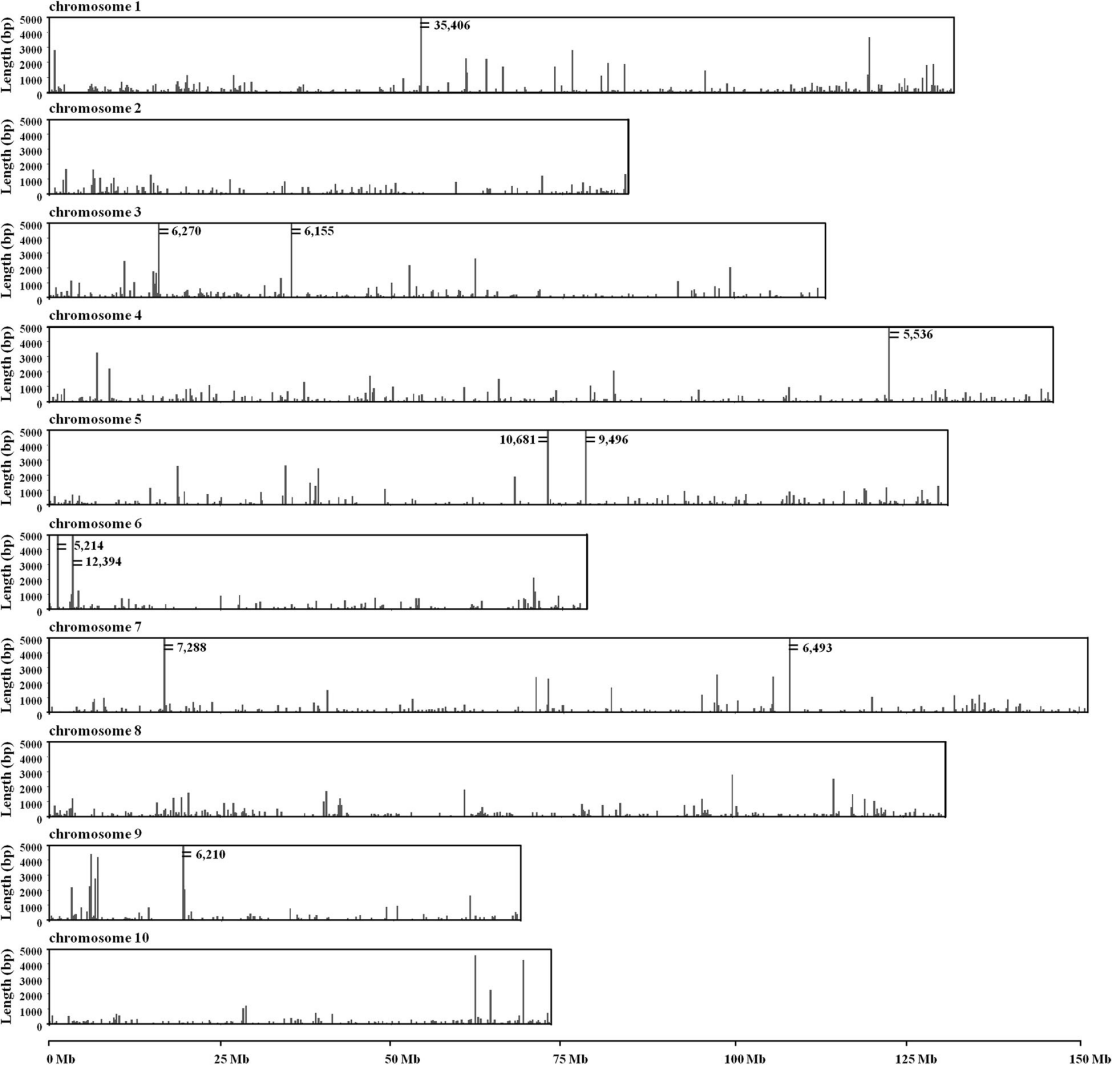
Distribution of NUPTs on 10 chromosomes of *A. officinalis*. The length of vertical lines indicates the length of the insertion, and each window size is 200 kb. Individual chromosomes are shown in separate panels

The distribution of NUPT sequences at different chromosomes and their positions were analyzed (Table 1, Fig. 2). Chromosome 1 (Y chromosome) presented the largest proportion of NUPTs and the largest number of detected NUPTs. A total of 440 NUPTs were localized on chromosome 1, accounting for 13.95% of the total NUPTs detected. The total length of NUPTs in chromosome 1 was 114,469 bp, accounting for approximately 0.09% of its size. Both the total and standard length of NUPTs (86,463 bp/100 Mb) of chromosome 1 were larger than those of other chromosomes. By contrast, chromosome 10 had the least NUPTs (169). However, considering the chromosome size, chromosome 4 was covered by the lowest proportion of NUPTs (~ 0.047%) (Table 1).

**Table 1:** NUPT sequences in the nuclear genome of *A. officinalis*

Chromosome	No. of NUPTs	Proportion of No. of NUPTs (%)	Density (No./100 Mb)	Total length of NUPTs (bp)	Standard length of NUPTs (bp/100 Mb)
1(Y)	440	13.95	332	114,469	86,463
2	234	7.42	276	42,048	49,626
3	314	9.95	277	71,867	63,453
4	344	10.90	235	69,160	47,189
5	382	12.11	291	82,497	62,750
6	187	5.93	237	50,734	64,400
7	377	11.95	249	80,276	53,036
8	379	12.01	289	70,305	53,529
9	190	6.02	277	46,566	67,940
10	169	5.36	230	35,123	47,819
Unplaced scaffolds	139	4.41	184	40,013	52,892
Total/average	3155	100	266	703,058	59,203

In the macroscopic scale, NUPTs were widely distributed throughout each chromosome (Fig. 2). However, the amount of NUPTs varied among different chromosomal regions. Some chromosomal regions clearly favor NUPT insertion. Within each sliding window of 200 kb, a total length of NUPTs > 5000 bp was found in 11 regions (Fig. 2) Chromosomes 3, 5, 6, and 7 each possessed two such regions, whereas chromosomes 1, 4, and 9 each had one such region. The highest NUPT coverage region was found within chromosome 1, where 35,406 bp sequences were detected as NUPTs within a 200 kb region. Detailed analysis of this region showed that NUPTs were mainly distributed in a region of less than 47 kb (locus site: 54519138–54,565,922). Within this 46,785-bp region, 35,328 bp sequences were detected as NUPTs.

**Fig. 3:**
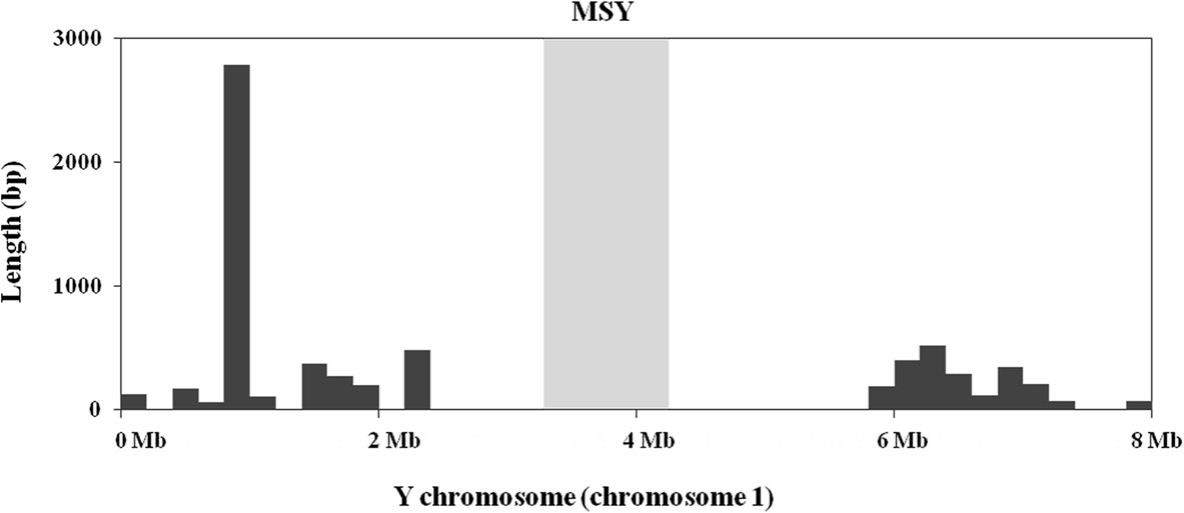
Distribution of NUPTs on the first 8 Mb of the Y chromosome. The MSY region is indicated by grey background

Interestingly, although the Y chromosome (chromosome 1) possessed the most number of NUPTs, the MSY (locus site: 3411433–4,258,465) and the adjacent regions did not show NUPT insertion (Fig. 3), which implies that NUPTs may not be involved in the formation of MSY in *A. officinalis*.

**Fig. 4:**
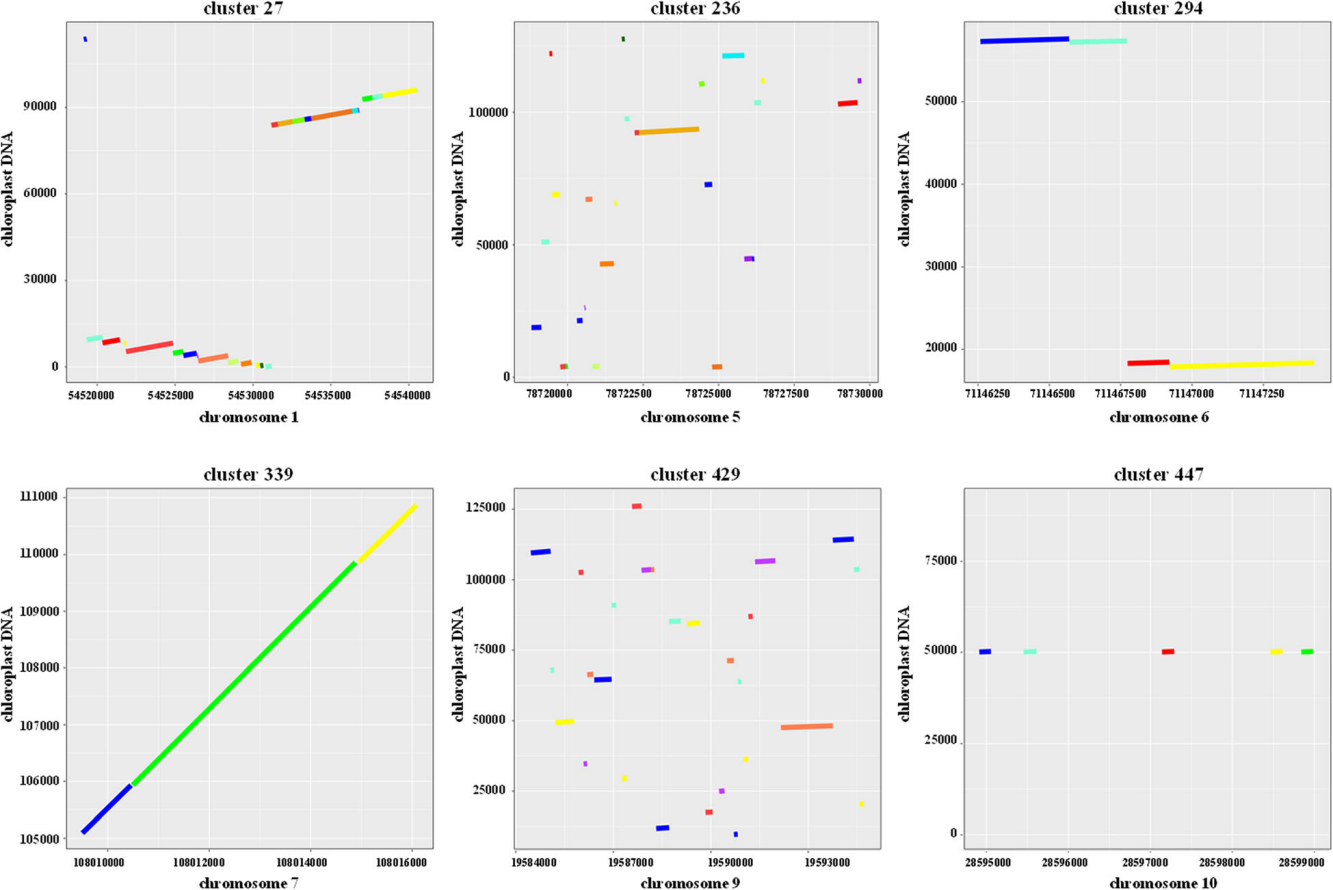
Dot-plot analysis of some typical NUPT clusters. NUPT cluster names are reported at the top of each plot. X axis reports the positions in the indicated chromosomes. Y axis reports the chloroplast sequence coordinates. Colored lines in the plots represent different NUPT sequences. Details about these regions are reported in Additional file 1: Table S1

The chromosome distribution analysis of NUPTs revealed that they were often clustered. We then conducted cluster analysis wherein two NUPTs are assigned to one cluster if they are separated by less than 5 kb. We found that 1396, or nearly 45% of the asparagus NUPTs were organized in such clusters (Additional file 1: Table S1). These NUPTs were assigned to 484 clusters, where the largest cluster (cluster 236) contained 26 NUPTs in chromosome 5. Another large cluster (cluster 27) had 25 NUPTs in chromosome 1 (Additional file 1: Table S1). Some typical clusters were visualized in dot-plots as shown in Fig. 4. NUPT clusters representing different insertion patterns were observed. These clusters were usually derived through unique insertional events, which underwent subsequent insertions, leading to separate, but tightly linked NUPTs identified as clusters (Fig. 4, cluster 339). The NUPT clusters were also frequently derived from several regions of the chloroplast DNA, each of which undergoes rearrangement with the evolution process (Fig. 4, clusters 27 and 294). We also identified some NUPT clusters organized by individual NUPT insertions, which originated from nearly identical chloroplast DNA region (Fig. 4, cluster 447). In addition, some NUPT clusters were derived from various regions of the chloroplast DNA, making these regions as chromosome hotspots of NUPT insertions (Fig.4, clusters 236 and 429).

### Distribution of NUPTs according to their origin from the chloroplast genome

**Fig. 5:**
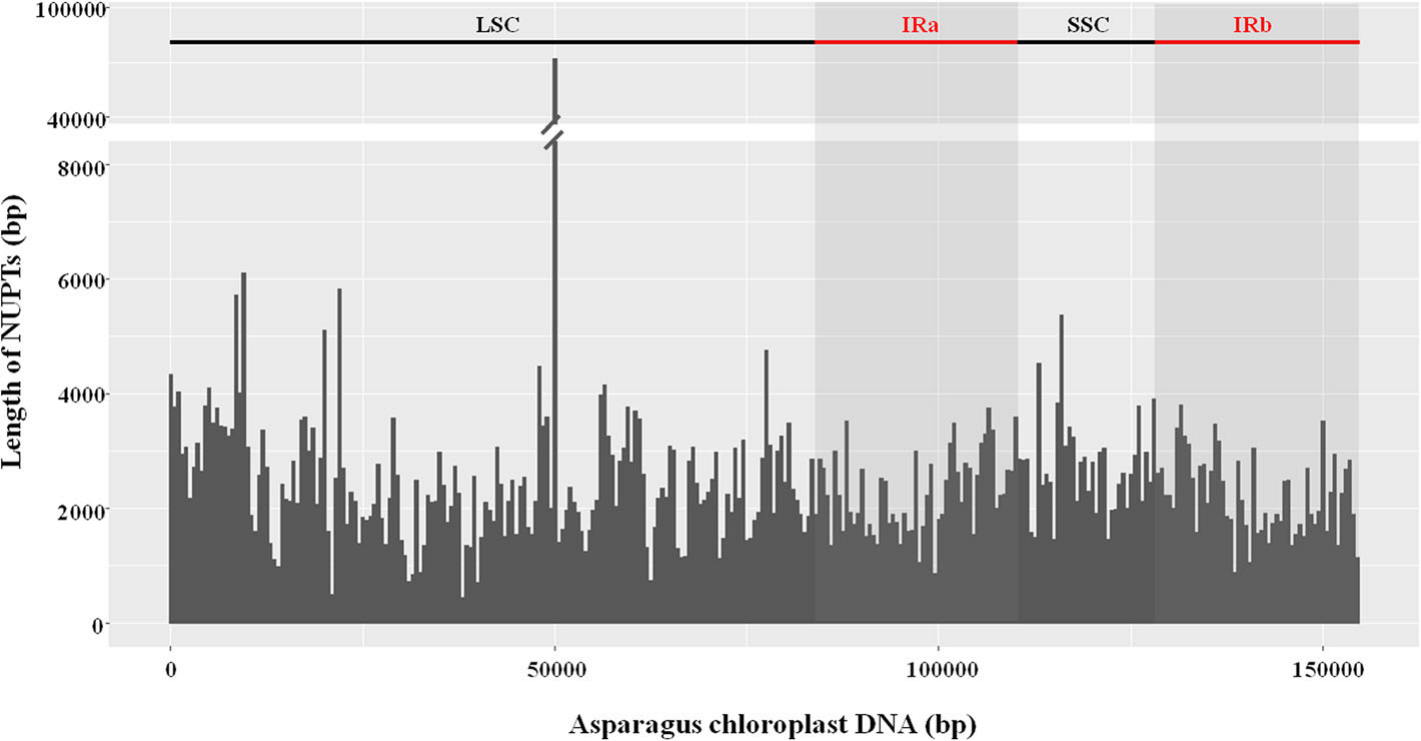
Distribution of NUPT sequences on the chloroplast genome. A sliding window of 500 bp was used to calculate for the total length of the inserted NUPTs. The two IR regions are marked by grey background

To understand the transfer pattern of chloroplast DNA into the nuclear genome of *A. officinalis*, we analyzed the origin of the NUPTs. Among the 3155 NUPTs, 571 were derived from inverted repeat (IR), 2166 from large single copy region (LSC), and 418 from small single copy region (SSC) (Table 2). The sizes of IR, LSC, and SSC were 52,942, 83,821, and 17,901 bp, respectively, while the densities of NUPTs originated from IR, LSC, and SSC were 11/kb, 26/kb, and 23/kb, respectively (Table 2). It is obvious that DNAs from the LSC region were more frequently transferred to the nuclear genome. Further analysis revealed that the high frequency of transfer from the LSC region was mainly due to a small region with a size of 286 bp (locus site: 50,003–50,288). A total of 443 NUPTs were derived from this region, accounting for 14% of the total number of NUPTs. The cumulative length of NUPTs originated from this region was 65,490 bp. Evidently, the sequences of the 286-bp region presented an unexpectedly high transfer frequency to nuclear genome (Fig. 5).

**Table 2:** Origin analysis of NUPT sequences on the chloroplast genome of *A. officinalis*

	IR	LSC	SSC
Total sequence length (bp)	52,942	83,821	17,901
Number of NUPTs	571	2166	418
Average density (No./Kb)	11	26	23
The length of NUPTs (bp)	136,080	472,807	94,171
Mean length of NUPTs	238	218	225
Mean *p*-distance	0.09	0.11	0.09

### Insertion age of NUPTs

**Fig. 6:**
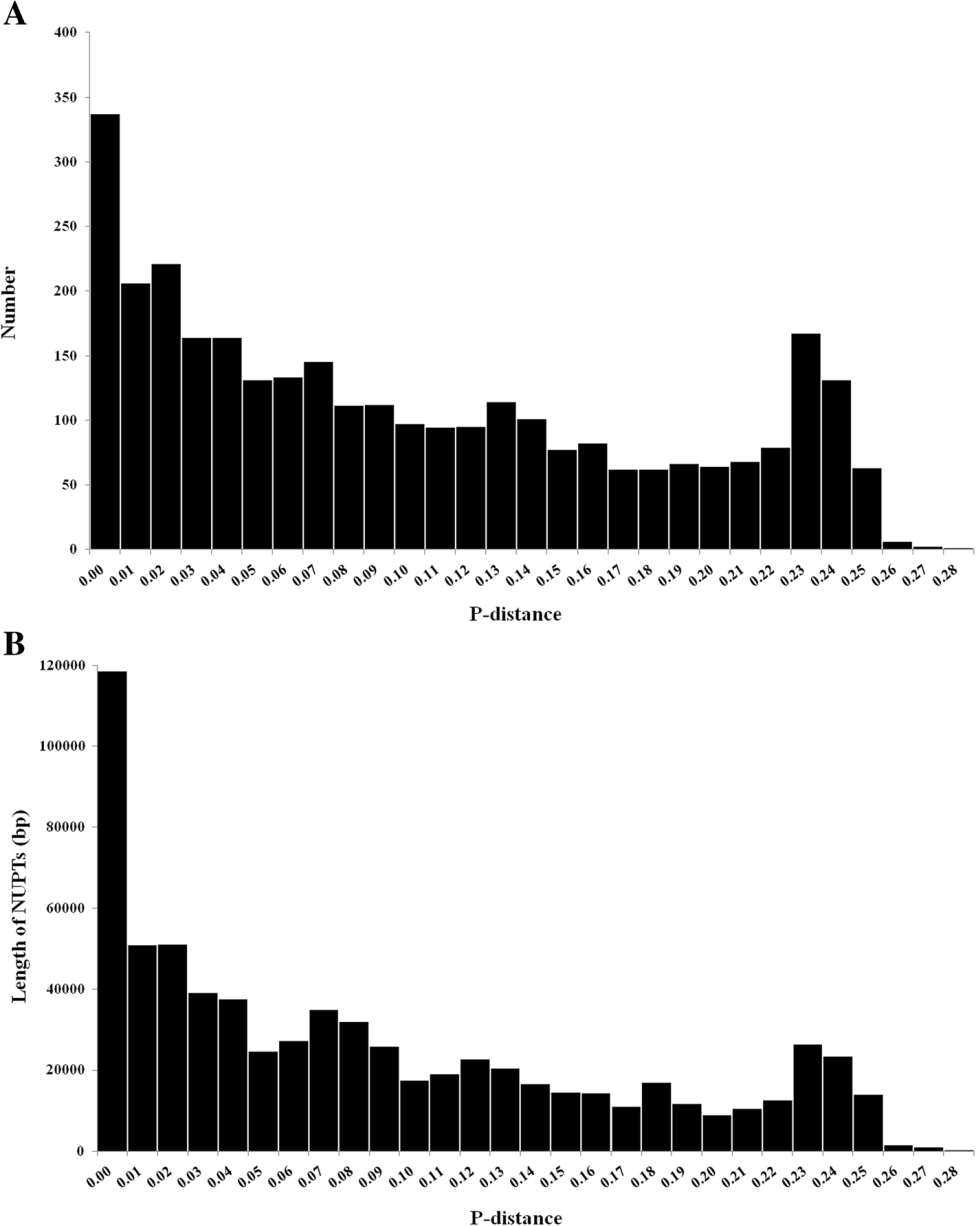
Age distribution of NUPTs in the nuclear genome of *A. officinalis*. The numbers (**a**) and cumulative length (**b**) of NUPTs for each *p*-distance interval are shown

We used the *p*-distance to calculate for the relative insertion age of each NUPT. Both for the number and the cumulative length of NUPTs had a high peak in the *p*-distance interval of 0–0.01 (Fig. 6), which represented the youngest age. Then, with the increase in *p*-distance, the number and cumulative length of NUPTs decreased, except that a small peak was observed in the *p*-distance interval of 0.23–0.25 (Fig. 6). This peak is mainly due to the high transfer frequency of the 286-bp sequence within the LSC region, because the majority of the NUPTs derived from the 286-bp region showed large *p*-distance from 0.23–0.25. Generally, the evolution time of NUPTs increased with the decrease in the length of NUPTs. The mean *p*-distance of NUPTs was 0.10, whereas that of NUPTs with length between 500 and 1000 bp was 0.07, and that of NUPTs longer than 1000 bp was only 0.05. The mean insertion age and mean length of NUPTs slightly differed among the different chloroplast DNA origins. The NUPTs that originated from the LSC region showed a mean *p*-distance of 0.11 and mean length of 218 bp. The mean insertion age of NUPTs derived from the IR and SSC regions was slightly low with a mean *p*-distance of 0.09. The average sizes of NUPTs derived from the IR and SSC regions were 238 and 225 bp, respectively (Table 2).

### Chromosome localization of NUPTs

A total of 32 pairs of primers were designed for overlapping chloroplast fragments with an average desired fragment length of approximately 4051 bp. Finally, 28 chloroplast fragments were amplified successfully. Among these fragments, 17, 8, and 3 belonged to the LSC, IR, and SSC region, respectively.

**Fig. 7:**
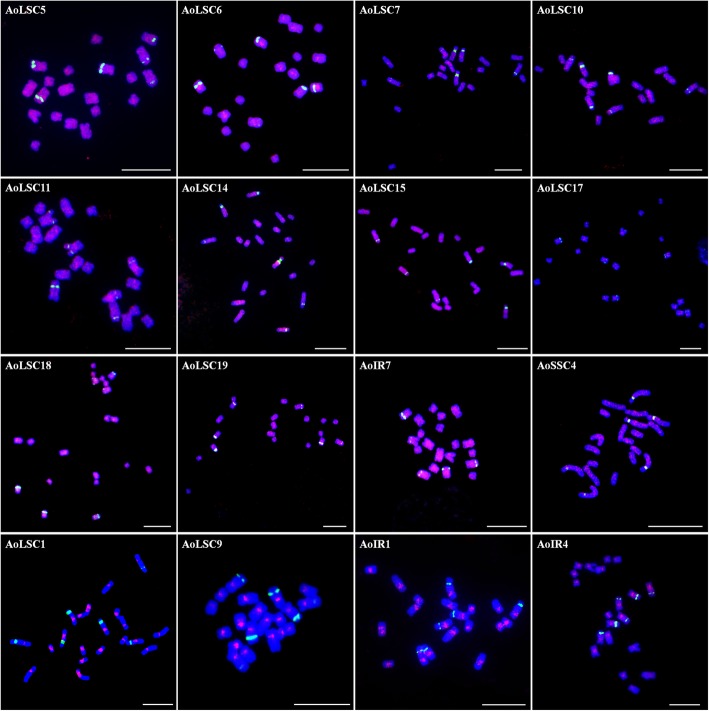
Chromosome localization of chloroplast DNA on the metaphase chromosomes of *A. officinalis* (signals widely distributed on all chromosomes). Each chloroplast DNA probe was labeled with Texas red (red signal), 45S rDNA was labeled with Chroma Tide Alexa Fluor 488 (green signal), and the chromosomes were counterstained with DAPI (blue). Bars = 10 μm

FISH analysis showed that 24 of the 28 probes could generate stable and clear signals on *A. officinalis* chromosomes. Among these, 12 probes were dispersed in almost all the chromosomes (Fig. 7). AoLSC5, AoLSC15, AoLSC18, AoLSC19, and AoIR7 showed evenly distributed signals on all the chromosomes, whereas AoLSC6, AoLSC7, AoLSC10, AoLSC11, AoLSC14, and AoSSC4 were more inclined to distribute in blocks along the chromosomes (Fig. 7). AoLSC17 showed weak signals mainly at the pericentromeric regions of all chromosomes. AoLSC1, AoLSC9, and AoIR1 mainly occupied the centromeric regions, whereas AoIR4 was distributed in the centromeric and adjacent pericentromeric regions of all the chromosomes (Fig. 7).

**Fig. 8:**
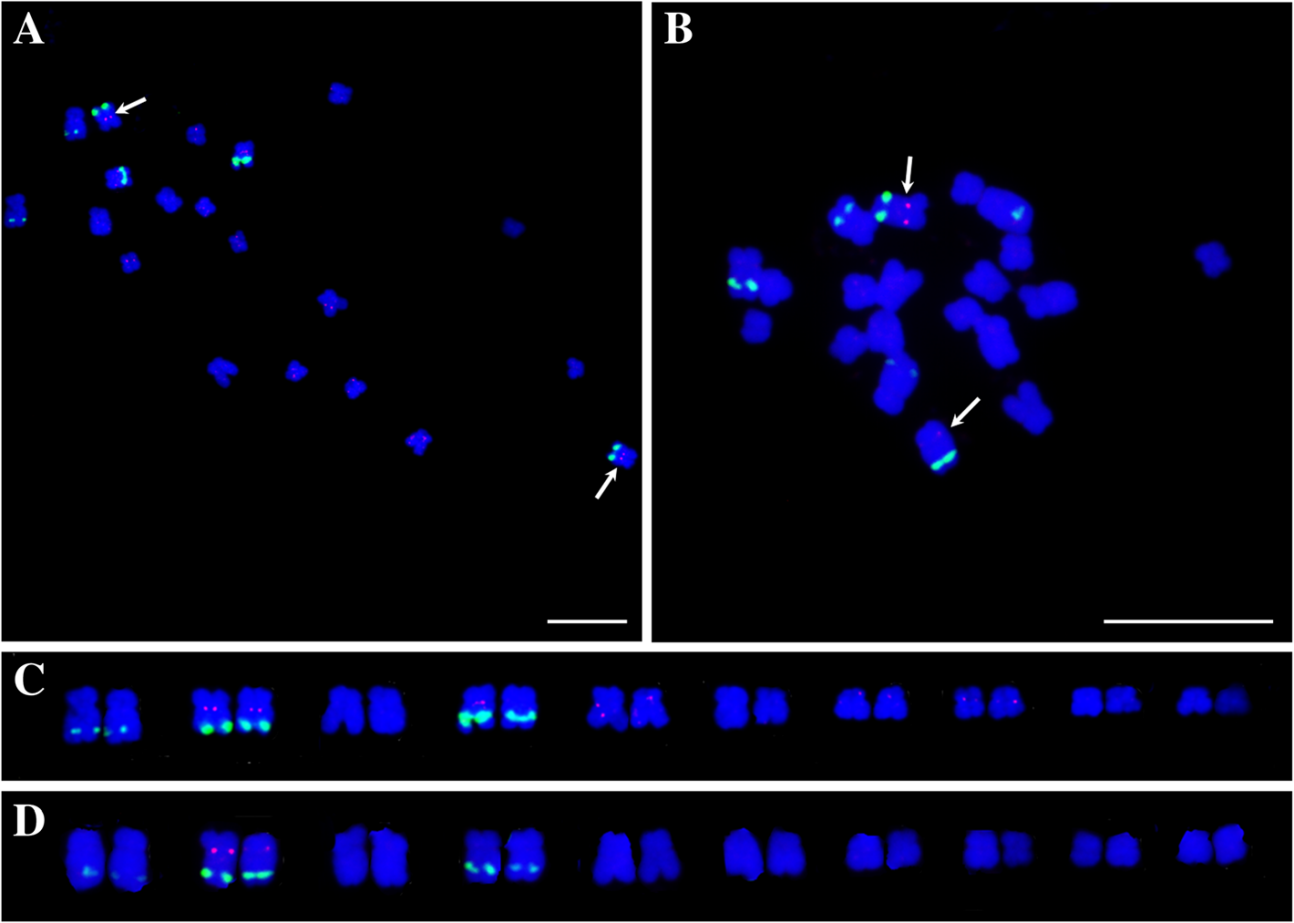
Chromosome localization of two chloroplast DNA fragments on metaphase chromosomes and karyotype analysis. **a** FISH result of AoLSC4; (**b**) FISH result of AoSSC3; (**c**) Karyotypic analysis of AoLSC4; (**d**) Karyotypic analysis of AoSSC3. Arrowheads indicate the sites of chloroplast DNA probe hybridization (red). Bars = 10 μm

The other eight probes showed more intense signals on specific chromosomes, among which, two probes (AoSSC3 and AoLSC4) were mainly distributed in the centromeric regions of chromosome 2 (Fig. 8). AoSSC3 was exclusively localized in the centromeric regions of chromosome 2 (Fig. 8a, c), while AoLSC4 was distributed in the other chromosomes, in addition to chromosome 2 (Fig. 8b, d).

**Fig. 9:**
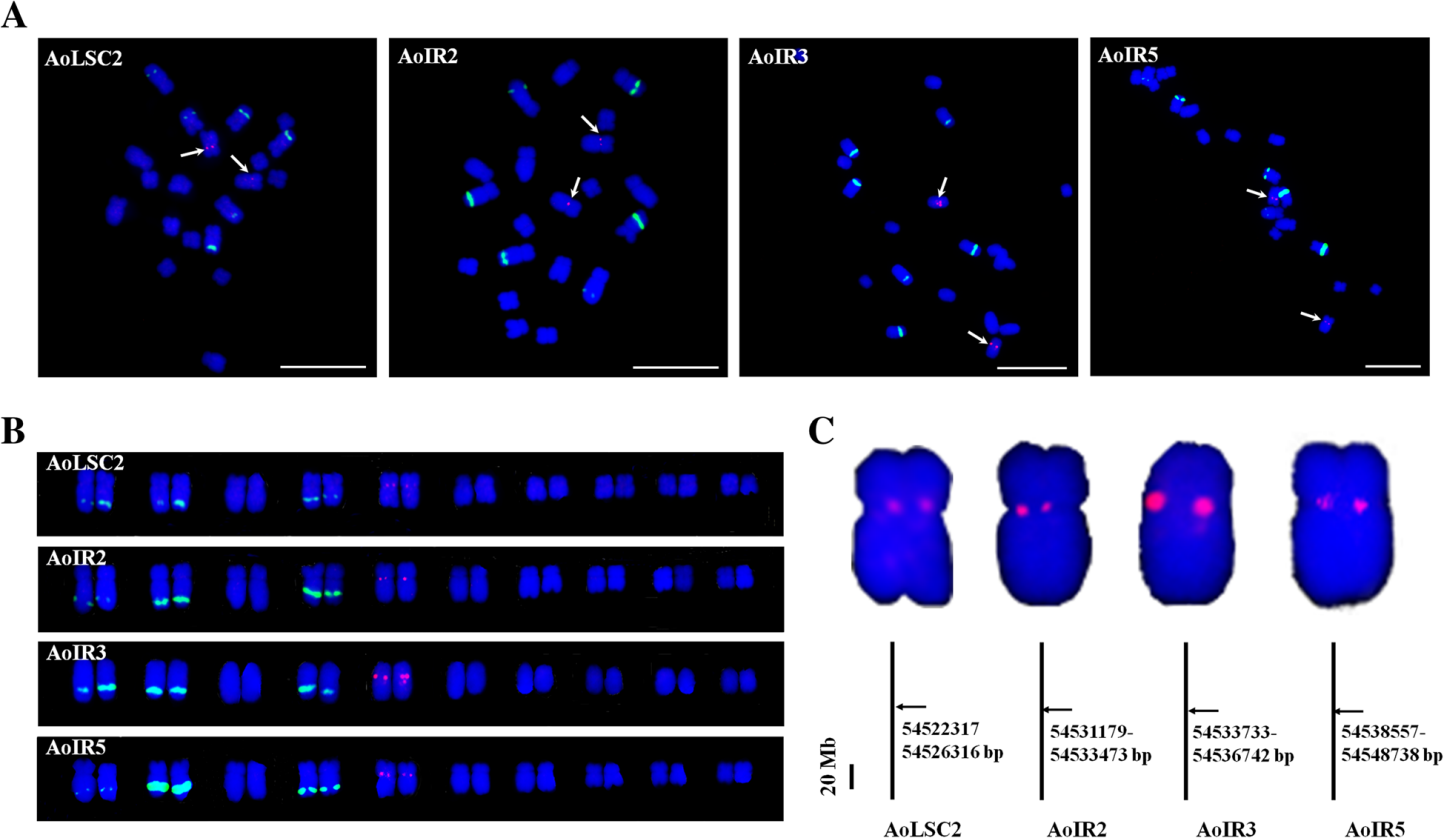
Cytogenetic and sequence alignment analysis of the four chloroplast DNA fragments localized on the centromeric regions of sex chromosomes. **a** FISH analysis of the four chloroplast DNA sequences. Arrowheads indicate the sites of chloroplast DNA probe hybridization (red). Bars = 10 μm; (**b**) Karyotypic analysis based on FISH results; (**c**) Enlarged sex chromosomes with FISH signals and BLAST analysis of these four chloroplast DNA sequences

More importantly, AoLSC2, AoIR2, AoIR3, and AoIR5 were all localized in the centromeric regions of one pair of chromosomes (Fig. 9a). Karyotype analysis shows that the four probes were localized on the same pair of chromosomes, i. e. the chromosome 5 (Fig. 9b). Previous studies reported that the pair of chromosome 5 in the karyotype analysis is the sex chromosome of *A. officinalis* [30, 31]. Genome sequence and analysis showed chromosome 1 of genome sequences is the sex chromosome. Thus, the chromosome 5 in the karyotype analysis is corresponding to chromosome 1 of the genome sequence. We also conducted BLAST analysis to investigate the distribution patterns of the NUPTs derived from the four chloroplast DNA fragments in the genome of *A. officinalis*. The results showed that NUPTs that originated from the four chloroplast DNA fragments were mainly distributed in adjacent regions in chromosome 1 of genome assembly (Fig. 9c). These sequences were concentrated on a region around 54–55 Mb of chromosome 1, which is within the region with maximum density of NUPTs, more than half of which is occupied by NUPTs (Fig. 2). Combined with the FISH results that these sequences were mainly located at the centromeric region of the sex chromosomes, the results suggested that the 54–55 Mb region should be within or around the centromeric region of the sex chromosome, and the centromeric regions of the sex chromosome of *A. officinalis* were occupied by plenty of NUPTs.

## Discussion

During evolution process, plant nuclear genomes have been shaped by the acquisition of new sequences of various origins. These sequences include TEs and the sequences derived from mitochondrial DNA (NUMTs) and cpDNA (NUPTs). TEs have been widely investigated in many plant genomes, and showed to play various roles in genome evolution, chromosome rearrangement, gene creation and regulation [32–36]. NUMTs have been studied extensively in animals [7, 37, 38]. By contrast, NUPTs only exist in plants and have been studied in a limited number of plant species [6, 31, 39]. In this study, systematic analyses and cytogenetic localization of NUPTs were conducted on a dioecious plant *A. officinalis*. The proportion of NUPTs in the asparagus genome (0.06%) is within the range observed in other plants. For example, the percentages of NUPTs in current analyzed nuclear genomes vary from 0.024% in *Sorgum bicolor* to 0.222% in *O. sativa* [40]. The NUPT proportion in the nuclear genome depends on the balance of the frequency of DNA migration from the plastids to the nuclear genome and the rate of NUPT loss in the nuclear genome [39]. It is also influenced by the assembly and annotation quality of the genome and the methods used in the analyses.

All the plastid DNA sequences can transfer to the nuclear genome of *A. officinalis*, indicating that all sequences are transferable. However, the transfer frequency from different parts of the plastid genome varied. The LSC region showed a high transfer rate, which is largely due to the 286-bp sequence with unexpectedly high transfer frequency. In most of the studied plant genomes, although the transfer rate varies among different regions of the chloroplast genome, no regions had an extremely low or high transfer frequency [40]. Thus, such high transfer frequency of a small size of region has not been reported in other species. Owing that nearly all the inserted NUPTs that originated from this 286-bp region showed similar and relatively large *p*-distance (ranging from 0.19–0.24 with only one exception), we speculate that these NUPT insertions were old-aged and may be formed through a sudden event caused by stress. In fact, experimental studies confirmed that stress can promote DNA migration from the chloroplasts to the nucleus. For example, DNA migration from chloroplasts to the nucleus is markedly increased by mild heat stress in two transplastomic tobacco lines [22].

Cluster analysis of NUPTs revealed that nearly 45% of the NUPTs were organized in clusters. Further dot-plot analysis indicated that these clusters might be formed through different evolutionary paths, implying complicated mechanisms shaping the genome of *A. officinalis*. A considerable number of NUPT sequences could be derived via a single or two insertional events that subsequently underwent mutations and chromosomal rearrangements. Mutations and chromosomal rearrangements can split the integrated cpDNA in separate, but co-linear regions. Such clusters derived from initially long cpDNA insertion and subsequent fragmentation have also been widely observed in organelle-derived insertions in other species [6, 41]. Long integrated NUPTs are assumed to be young and recent insertions, whereas short fragments are usually old insertions [42–44]. The relative age analyses results of NUPTs in *A. officinalis* are consistent with this assumption. In addition, the assumption is well supported by the co-linear cluster regions observed widely in the genome of *A. officinalis*. Long NUPTs might be degraded and fragmented by mutations and chromosomal rearrangements with the ongoing evolutionary process. NUPTs organized in clusters were also frequently derived from different regions of the chloroplast DNA. These chromosome regions recruited a large number of NUPT sequences, but no co-linear pattern was investigated. Such complicated regions might be formed through various direct NUPT insertions, implying that these regions could be more attractive for cpDNA integration. This phenomenon was probably due to the features of the chromosomal regions themselves, such as the condensation state of the region. For example, the pericentromeric regions which usually presenting dense heterochromatin state are hotspots for organelle-nucleus DNA transfer [15]. Alternatively, these regions might be formed by genome shuffling through chromosome rearrangement.

NUPTs are distributed unevenly within the chromosomes of *A. officinalis*. Some regions prefer NUPT insertion. We discovered a 47 kb region, where more than 75% was occupied by NUPTs. This biased distribution pattern was also discovered in other analyzed plant genomes. For example, some chromosomal regions that are rich in NUPTs were found in *Lotus japonicus*, *O. sativa*, and *Zea mays* [40]. It has been reported that NUPTs are frequently distributed in pericentromeric regions, especially in species with a small genome size, whereas NUPTs have a wide distribution pattern in species with large genome sizes [6]. The location of the centromeres was unknown in the assembled chromosomes of *A. officinalis*. However, FISH analysis revealed that four chloroplast DNA sequences were preferentially distributed in the centromeric and adjacent regions of all the chromosomes. In addition, four fragments were exclusively located in the centromeric regions of the sex chromosomes, and two other sequences mainly occupied the centromeric regions of chromosome 2. These findings suggested that NUPTs likely more accumulated in centromeric or pericentromeric regions in *A. officinalis*. Centromeric and pericentromeric regions are hotspots of organelle–nucleus DNA transfer [15, 39], because of the low gene density and high heterochromatin content of these regions [43–46], which may provide a stable genomic environment for the integration of the organelle-derived DNA [6, 15]. However, Yoshida et al. showed that NUPTs also accumulate in the distal regions of chromosomes [38]. This finding might imply that in addition to pericentromeric regions, other regions also have high tolerance to NUPTs.

Both bioinformatic and cytogenetic analyses showed that the sex chromosomes of *A. officinalis* contained more NUPTs. However, the MSY and adjacent regions did not have NUPT insertion. These results indicated that NUPTs might facilitate the shaping of sex chromosome, but were not involved in the MSY evolution. Studies have shown that NUPTs preferentially accumulate on the sex chromosomes of *R. acetosa*, *S. latifolia*, and *C. papaya* [23–25]. These transferred organellar DNA sequences are possibly one of the driving forces in the sex chromosome evolution of dioecious plants. It is universally believed that sex chromosomes are derived from pairs of autosomes [47]. During the evolution process, sex chromosomes can recruit a large number of TEs and organellar DNAs [25, 48], and these sequences may facilitate the structural evolution of sex chromosomes. For example, the inserted TEs and organellar DNAs may lead to structural and morphological differentiation of sex chromosomes, and contribute to the degeneration of Y chromosomes [49]. However, the involvement of NUPTs in the MSY formation is poorly studied. In *C. papaya*, NUPTs that accumulate in HSY and MSY are much more than those in the X chromosome and the autosomes [25]. Furthermore, organellar DNA insertions are sparse between X and HSY, suggesting that the organellar DNA accumulates after the recombination of HSY is suppressed. The accumulation of NUPTs in papaya has contributed to the dramatic expansion and repeated accumulation in HSY [25]. In this study, NUPTs did not accumulate in the MSY of *A. officinalis*, indicating that NUPTs did not contribute to the formation and recombination suppression of MSY in this dioecious plant. The sex chromosomes of *A. officinalis* are very young. These results are consistent with the findings in papaya, whose NUPT insertions occur after the HSY is suppressed for recombination. However, the sex chromosomes do harbour more NUPTs than the autosomes. The mechanism and function of these NUPT insertions in the recombining regions of sex chromosomes are still not clear currently. One reasonable explanation is that although the sequences outside the MSY are usually not related to sex determination, they also suffered sexual selection because of linkage disequilibrium and may show different evolutionary rate compared with the sequences on the autosomes. In fact, the mechanism of sex determination and sex chromosome evolution may be far more complex than traditionally appreciated and usually unpredictable. For example, the recent discovered gene *MpFGMYB*, which encodes a key regulator of female sexual differentiation in liverwort, localized in autosomes, not sex chromosomes [50]. Full understanding of these processes involved in sex determination and sex chromosome evolution need further studies and accumulation of more data. Currently, the X chromosome of *A. officinalis* has not been sequenced. In the future, with the sequence of the X chromosome available, a comparison between the sequences of the X and Y chromosomes can be conducted. More information about the early stages of sex chromosome evolution can be obtained.

## Conclusion

In conclusion, this study presented a comprehensive bioinformatics and cytogenetic analysis of NUPTs in the nuclear genome of *A. officinalis*, an important vegetable and model dioecious plant species for studying the early stages of sex chromosome evolution. More than 45% of the NUPTs were organized in tight clusters, mainly including separate but co-linear clusters and promiscuous insertional clusters with no co-linear pattern. The large differences among the NUPT origins suggest that the integrated regions might be formed through different evolutionary paths and NUPTs were involved in shaping the genome of *A. officinalis* through complicated mechanisms. NUPTs preferably accumulated on the sex chromosomes of *A. officinalis* but not at the MSY. Hence, NUPTs might play a role in the shaping of sex chromosome structure but were irrelevant to the MSY formation. Our analysis provided new insights into the genome structure and evolution of *A. officinalis* and established a basis for further studying the sex chromosome evolution of *A. officinalis*.

## Methods

### Plant materials

*A. officinalis* cultivar ‘UC309’ grown in the garden field of Henan Normal University was used for the experiments. The original seeds were bought from Henan Yinong Company in 2005. One male and one female plant generated from the seeds were crossed to produce F_1_. The F_2_ individuals generated from the F_1_ and the corresponding seeds were used in this study.

### Data sources

The nuclear genome of *A. officinalis* was downloaded from https://www.ncbi.nlm.nih.gov/genome/?term=10978 [28], and the sequence of the chloroplast genome was provided by Dr. Pires [51]. The sizes of the nuclear and chloroplast genomes were 1187.54 Mbp and 154,664 bp, respectively.

### Detection of NUPTs

NUPT insertions were identified using the BLASTN local alignment tools in the BLAST program package (ver. 2.2.31) with chloroplast genomic DNA as the query sequence and nuclear genome data as the database. The parameters were as follows: -dust no, *e*-value threshold of 1e-4, mismatch penalty of − 2, and word size of 9. The BLAST hits for the NUPTs that originated from the IR region of the chloroplast genome were counted only once because these BLAST hits were obtained in both IR regions and could not be distinguished. The NUPTs distributed on the nuclear chromosomes and originating from the cpDNA were visualized based on slide window lengths of 200 kb and 500 bp, respectively.

### Estimation of NUPT age distribution

The *p*-distance between each NUPT and the corresponding chloroplast DNA sequence was calculated to estimate the relative integration time. *p* = *n*_*d*_/*n*, where *n*_*d*_ is the number of nucleotide differences, and *n* is the number of aligned nucleotides [10].

### PCR amplification of cpDNA regions

The total genomic DNA was isolated from young leaves of *Asparagus officinalis*. To obtain overlapping fragments representing the entire chloroplast genome of *A. officinalis*, the primers were designed using OLIGO 7 program [52]. The primer sets are listed in Additional file 2: Table S2. A total of 32 chloroplast DNA PCR products were amplified from the total extracted genomic DNA, containing the chloroplast DNA. The PCR amplification fragments were verified by sequencing the 5′- and 3′-end sequences.

### FISH procedure

Slide preparation using mitotic metaphase spreads from the root tips, probe-labeling, hybridization, and picture-processing were done as previously described [53]. The individual chloroplast regions were labeled with Texas Red-5-dCTP (shown in red) and hybridized at a final concentration of 20 ng/μL each. The 45S rDNA was labeled with fluorescein-488-dUTP (shown in green), and double-color FISH was performed for a better characterization of the chromosomes. After hybridization and washing, all slides were treated with Vectashield (Vector Laboratories, Burlingame, Calif.) containing 4′,6-diamidino-2-phenylindole (DAPI) prior to visualization.

## Additional files

Additional file 1: **Table S1.** The detailed information of NUPTs in the nuclear genome of *A. officinalis*. (DOCX 44 kb)

Additional file 2: **Table S2.** The primers used for amplification of chloroplast DNA fragments of *A. officinalis*. (XLSX 278 kb)

## Data Availability

The nuclear genome of *A. officinalis* was downloaded from https://www.ncbi.nlm.nih.gov/genome/?term=10978. The data and plant material that support the findings of this study are available from the corresponding author on request. All data generated or analyzed during this study are included in this published article.

## Abbreviations

BAC: Bacterial artificial chromosome
cpDNA: chloroplast DNA
DAPI: 4′,6-diamidino-2-phenylindole
FISH: Fluorescence in situ hybridization
HSY: Hermaphrodite-specific regions of Y^h^
IR: Inverted repeat
LSC: Large single copy region
MSY: Male-specific region of the Y chromosome
NUMTs: Nuclear integrants of mitochondrial DNAs
NUPTs: Nuclear integrants of plastid DNAs
SSC: Small single copy region
TEs: Transposable elements
