# Supplementary material for: Cytogenetic and genomic organization analyses of chloroplast DNA invasions in the nuclear genome of Asparagus officinalis L. provides signatures of evolutionary complexity and informativity in sex chromosome evolution
Source: BMC Plant Biol. 2019 Aug 16;19:361. doi: 10.1186/s12870-019-1975-8 (PMC6698032; doi:10.1186/s12870-019-1975-8)
Supplement: Supplementary file 1 — Table S1. The detailed information of NUPTs in the nuclear genome of A. officinalis. (DOCX 44 kb) [file 12870_2019_1975_MOESM1_ESM.docx]

TABLE S1. *The primers used for amplification of chloroplast DNA fragments of garden asparagus*

| Name | Primer sequence (5′-3′) | Sequence length (bp) | Annealing temperature (°C ) | Region |
| --- | --- | --- | --- | --- |
| Aocp1/LSC1 | F: CAAGATATTGGGCATGGCTC | 3994 | 55 | LSC |
|  | R: ACTCAACGGTAGAGTACTCG |  |  |  |
| Aocp2/LSC2 | F: TACTCTACCGTTGAGTTAGCA | 3851 | 55 | LSC |
|  | R: ATTCTGATTCCTCCATTACC |  |  |  |
| Aocp3/LSC3 | F: CAAGGACTTTCTAAGGACCC | 3998 | 55 | LSC |
|  | R: AGAGCGATTAATCAGGTCCG |  |  |  |
| Aocp4/LSC4 | F: GAATCCAGTACCGACAGGG | 3896 | 58 | LSC |
|  | R: ATAGATCATTTCGGAATGGC |  |  |  |
| Aocp5/LSC5 | F: TGAAATAGATGTAGCAGTGG | 4040 | 55 | LSC |
|  | R: GTTATTGATGTGCGATGGA |  |  |  |
| Aocp6/LSC6 | F: TGGATTATAACTGGAACCC | 4013 | 55 | LSC |
|  | R: GACCTTAGTTCTGAATCCC |  |  |  |
| Aocp7/LSC7 | F: GGTATCCAAAGGTTACTCC | 4126 | 57 | LSC |
|  | R: GGTAAACACGATTCACTTGG |  |  |  |
| Aocp8/LSC8 | F: GGATTCTACCCTGTAGTGAC | 4006 | 55 | LSC |
|  | R: AGCAAATACAACGGGTACAC |  |  |  |
| Aocp9/LSC9 | F: GGCAACCACCATTATAACGA | 3870 | 55 | LSC |
|  | R: TTATTCGTGGCATGGAGTCA |  |  |  |
| Aocp10/LSC10 | F: ACTTCCTTTGACTCCATGC | 4255 | 55 | LSC |
|  | R: GGGACATGTCAAGTATCCG |  |  |  |
| Aocp11/LSC11 | F: TGTACATCCAGAATAGACCT | 4070 | 55 | LSC |
|  | R: TTACACCTATGGGTTCTGC |  |  |  |
| Aocp12/LSC12 | F: TTGATTCCTAGTGTAGTGC | 4305 | 55 | LSC |
|  | R: TCTTAAACGTATCTTGATGCC |  |  |  |
| Aocp13/LSC13 | F: CGATACGGATTAGAGTCCAG | 4060 | 55 | LSC |
|  | R: TTAGGTAGAGCACCTCGTT |  |  |  |
| Aocp14/LSC14 | F: CATGATAGCTTTATGATCCG | 4156 | 55 | LSC |
|  | R: AGCCACGGGAATAACACC |  |  |  |
| Aocp15/LSC15 | F: TCTATGCCAGGTGTTATTCC | 3968 | 57 | LSC |
|  | R: CAAGAAATAGGCCAATTCGG |  |  |  |
| Aocp16/LSC16 | F: GTCGCATCTTCCTTCGCTTC | 4266 | 57 | LSC |
|  | R: TTTCACAGAGAGCCGACAGG |  |  |  |
| Aocp17/LSC17 | F: CGGATAGGTTCGATCTATGG | 4012 | 58 | LSC |
|  | R: AGTTCTAGGGATCGACTCGG |  |  |  |
| Aocp18/LSC18 | F: TTGACCTTGAAACAACAACG | 4166 | 55 | LSC |
|  | R: GTAAGAACAAAGAGAAGCAG |  |  |  |
| Aocp19/LSC19 | F: ATCCATGCCTTTGTTCCC | 4079 | 55 | LSC |
|  | R: CTTGCTTACGTATCATTGGG |  |  |  |
| Aocp20/LSC20 | F: ATTGCCTCTTCTTACTGC | 3929 | 57 | LSC |
|  | R: ATATGGTGATCCTCCTCC |  |  |  |
| Aocp21/IR1 | F: CTAGCTTCTCGATCTGTCA | 4140 | 57 | IR |
|  | R: TCCACTTGGCTACATCCG |  |  |  |
| Aocp22/IR2 | F: GATCTCAATTCCAGCTACGG | 3614 | 55 | IR |
|  | R: GTAGGTATCTGGTTCACTGC |  |  |  |
| Aocp23/IR3 | F: GATATTTGCACCCTGTCG | 4254 | 55 | IR |
|  | R: TGTCCCATAATAGGTCCC |  |  |  |
| Aocp24/IR4 | F: AGATAGGAAGGGCTGTTGC | 4118 | 55 | IR |
|  | R: AGCAGGGTCAGGAACAACG |  |  |  |
| Aocp25/IR5 | F: TTCGGAAACCCAAGGACT | 4042 | 55 | IR |
|  | R: AATTCCAACAGCTCGGAC |  |  |  |
| Aocp26/IR6 | F: CATCTTGACTTGTTCTGCT | 3950 | 55 | IR |
|  | R: CTACGGGGTGGAGACGATG |  |  |  |
| Aocp27/IR7 | F: CTTTCTTCCGTTTCTGAGG | 4029 | 55 | IR |
|  | R: GAAAGGCGTAGTCGATGGA |  |  |  |
| Aocp28/IR8 | F: TGCAAAGGTCGTCTTATTC | 3989 | 55 | IR |
|  | R: TACTACTGTTGGAATCATGG |  |  |  |
| Aocp29/SSC1 | F: ACACATTCCTACAAGTTCC | 4280 | 55 | SSC |
|  | R: CAGCTTGGCCTGTTACTCG |  |  |  |
| Aocp30/SSC2 | F: GTACATTGCTAACATCAGG | 4247 | 58 | SSC |
|  | R: CAAGCAGTACTTCCTCACG |  |  |  |
| Aocp31/SSC3 | F: TCAGCGAATAGGAGCATAC | 4068 | 55 | SSC |
|  | R: AAAGATCGGCCTAAATCAC |  |  |  |
| Aocp32/SSC4 | F: TGTCTGCTCCCGTGTAATC | 3850 | 55 | SSC |
|  | R: AGGAACAAGAGGGATCCAC |  |  |  |
